# The Role of Plasma and Urine Metabolomics in Identifying New Biomarkers in Severe Newborn Asphyxia: A Study of Asphyxiated Newborn Pigs following Cardiopulmonary Resuscitation

**DOI:** 10.1371/journal.pone.0161123

**Published:** 2016-08-16

**Authors:** Daniel Sachse, Anne Lee Solevåg, Jens Petter Berg, Britt Nakstad

**Affiliations:** 1 Department of Medical Biochemistry, Institute of Clinical Medicine, University of Oslo, Oslo, Norway; 2 Department of Chemistry, University of Oslo, Oslo, Norway; 3 The Department of Paediatric and Adolescent Medicine, Akershus University Hospital, 1478 Lørenskog, Oslo, Norway; 4 Department of Medical Biochemistry, Oslo University Hospital, Oslo, Norway; 5 Institute of Clinical Medicine, Campus Akershus University Hospital, University of Oslo, Oslo, Norway; University of Nebraska Medical Center, UNITED STATES

## Abstract

**Background:**

Optimizing resuscitation is important to prevent morbidity and mortality from perinatal asphyxia. The metabolism of cells and tissues is severely disturbed during asphyxia and resuscitation, and metabolomic analyses provide a snapshot of many small molecular weight metabolites in body fluids or tissues. In this study metabolomics profiles were studied in newborn pigs that were asphyxiated and resuscitated using different protocols to identify biomarkers for subject characterization, intervention effects and possibly prognosis.

**Methods:**

A total of 125 newborn Noroc pigs were anesthetized, mechanically ventilated and inflicted progressive asphyxia until asystole. Pigs were randomized to resuscitation with a FiO_2_ 0.21 or 1.0, different duration of ventilation before initiation of chest compressions (CC), and different CC to ventilation ratios. Plasma and urine samples were obtained at baseline, and 2 h and 4 h after return of spontaneous circulation (ROSC, heart rate > = 100 bpm). Metabolomics profiles of the samples were analyzed by nuclear magnetic resonance spectroscopy.

**Results:**

Plasma and urine showed severe metabolic alterations consistent with hypoxia and acidosis 2 h and 4 h after ROSC. Baseline plasma hypoxanthine and lipoprotein concentrations were inversely correlated to the duration of hypoxia sustained before asystole occurred, but there was no evidence for a differential metabolic response to the different resuscitation protocols or in terms of survival.

**Conclusions:**

Metabolic profiles of asphyxiated newborn pigs showed severe metabolic alterations. Consistent with previously published reports, we found no evidence of differences between established and alternative resuscitation protocols. Lactate and pyruvate may have a prognostic value, but have to be independently confirmed.

## Introduction

Globally, perinatal asphyxia is one of the leading causes of morbidity and mortality in the neonatal period [1] and prompt and effective resuscitation is required to prevent neurological damage. Key parameters in neonatal resuscitation, such as whether to ventilate with air or pure oxygen during chest compressions (CC), the duration of initial assisted ventilation before initiation of chest CC, or the CC: ventilation (C:V) ratio have not been thoroughly studied. The efficacy of different protocols for cardiopulmonary resuscitation (CPR) is difficult to study in human infants for ethical reasons. Therefore, we have previously investigated the clinical outcomes of different CPR protocols in asphyxiated newborn pigs [2–5], and found reassuringly little evidence that any of the protocols performed significantly worse than others. In particular, air was as effective as pure oxygen in resuscitation from asystole, as were different C:V ratios. Ventilation periods of 30 and 60 s performed equally well, however, prolonging initial ventilation to 90 s led to significantly longer time to return of spontaneous circulation (ROSC) [2].

Metabolomics is an approach to quantitate a large number of small metabolites with molecular weight typically below 1000 Da within cells, tissues or biological fluids. The metabolites comprising the metabolome are end products of cellular activities and give a snapshot of physiological and pathophysiological processes. During asphyxia and resuscitation normal homeostasis is severely disturbed with profound effects on metabolism [6]. Previous studies of metabolic profiles during resuscitation of newborn piglets have demonstrated that the use of pure oxygen is associated with biochemical markers of delayed cellular recovery and increased oxidative stress [7–9]. Studies on newborn infants or cord blood have also described metabolomic alterations related to asphyxia and hypoxic ischemic encephalopathy [10–14].

The aim of this study was to analyze the impact of asphyxia and the different CPR protocols described in [2–5] on the metabolism of newborn pigs as measured by untargeted metabolite profiling of urine and plasma. Specifically, we searched for differences in the response to the alternative CPR protocols, as well as for biomarkers that can predict the individual outcomes. Furthermore, the study sought to characterize the metabolic trajectory over the course of the experiments and the correlation between corresponding plasma and urine samples.

## Materials and Methods

### Experimental Protocol and Sample Collection

The experimental protocol has been described in detail elsewhere [2–5]. The experimental protocol was approved by the Norwegian Council for Animal Research (Permit number: 13/08-845). Animals were cared for and handled in accordance with the European Guidelines for Use of Experimental Animals by certified category C researchers of the Federation of European Laboratory Animals Science Associations. The entire experiment was performed under fentanyl and midazolam anesthesia, and all efforts were made to minimize suffering. If signs of pain and/or distress, a bolus of fentanyl (50 μg/kg) and midazolam (2.5 mg/kg) was given.

Briefly, 125 healthy newborn Noroc pigs, aged 14–36 h (1.8–2.7 kg), were anesthetized with 5% Sevoflurane gas prior to a bolus of intravenous midazolam, fentanyl and pentobarbital. The animals were tracheotomized, placed on a mechanical ventilator, and intravenous access in the left external jugular vein was established. Each piglet was allowed to recover/stabilize for one hour prior to asphyxiation, and heart rate, arterial blood pressure, and transcutaneous oxygen saturation were monitored continuously. Clinical assessment with regards to e.g. pain and seizures was performed continuously. There were no unexpected deaths. The only deaths prior to the study endpoint at 4 hours after ROSC were caused by unsuccessful resuscitation, which was expected as per protocol. Even though the protocol dictated euthanasia if persistent signs of pain despite bolus analgesia or clinical seizures, no animals had to be euthanized before the study endpoint according to these criteria. This was not a survival study, but an acute experiment, meaning that the animals were euthanized with 150 mg/kg pentobarbital at a predetermined time point (4 hours after ROSC). This decision was partly made based on an expectation that having the animals surviving the experiment would be unethical as the animals would have suffered large neurological injury.

Baseline urine and blood samples were collected after stabilization, immediately before initiation of asphyxiation (t1). Then progressive asphyxia was introduced by adding CO_2_ to inspired air and reducing FiO_2_ to 0.08. The ventilator rate was reduced by 10 min^–1^ every 10 min until asystole occurred. After 20 s of asystole, the animals were resuscitated using six different, randomly assigned protocols [2–5] (Table 1). The varying number of pigs in each group is due to pooled experimental series. Samples were collected 2 h (t2) and 4 h (t3) after ROSC, defined as a heart rate ≥100bpm.

**Table 1:** Parameters of the six included resuscitation protocols.

Group	In.V.^a^	Oxygen	C:V ratio^b^	Time to asystole^c^	Time to ROSC^d^
1 (n = 21)	30 s	100%	3:1	28.0 (24.0–35.0)	135 (121–168)
2 (n = 32)	30 s	21%	3:1	32.5 (27.8–35.5)	146 (125–173)
3 (n = 21)	60 s	21%	3:1	32.0 (29.0–35.0)	170 (146–184)
4 (n = 8)	90 s	21%	3:1	33.0 (30.3–36.0)	250 (202.5–330)
5 (n = 16)	30 s	21%	9:3	35.0 (30.8–40.3)	145 (118–173)
6 (n = 12)	30 s	21%	15:2	37.0 (33.5–39.0)	195 (155–315)

^a^Initial Ventilation.

^b^Compression to ventilation ratio.

^c,d^Time in seconds (median and IQR) to asystole and return of spontaneous circulation (ROSC), respectively. The remaining 15 animals were resuscitated using varying parameters and are excluded in most analyses.

Urine samples were immediately frozen in liquid nitrogen and stored at -70°C, whereas EDTA blood was centrifuged and the supernatant frozen at -70°C. Table 2 shows the number of available urine and plasma samples at the three time points.

**Table 2:** Number of available samples.

Group	Plasma t1 (n)	Urine t1 (n)	Plasma t2 (n)	Urine t2 (n)	Plasma t3 (n)	Urine t3 (n)
0^a^ (n = 15)	14	7	13	4	2	1
1 (n = 21)	20	18	21	16	17	7
2 (n = 32)	32	29	28	24	25	12
3 (n = 21)	21	17	20	13	15	9
4 (n = 8)	8	7	8	5	7	4
5 (n = 16)	16	16	16	15	15	7
6 (n = 12)	11	11	8	8	9	8
**Sum 1–6**	**108**	**98**	**101**	**81**	**88**	**47**

Available samples of pooled experimental series of alternative protocols of resuscitation at baseline (t1), 2 h (t2) and 4 h (t3) after return of spontaneous circulation (ROSC).

^a^15 animals were resuscitated using varying parameters and are excluded in most analyses.

### Acquisition of spectra

Plasma and urine samples were thawed at room temperature. Plasma samples were centrifuged at 2,000 g and 4°C for 10 min to precipitate debris that had not been completely removed immediately after sample collection. Two hundred and fifty μL of each sample were then mixed directly in 5 mm tubes (Wilmad LabGlass, Vineland, NJ, USA) with 250 μL NaH_2_PO_4_/NaOH buffer at pH 7.4 in 10% D_2_O and NaN_3_ containing deuterated trimethylsilylpropionate (TSP) as a frequency and concentration reference.

For urine, 550 μL of each sample were buffered with 55 μL KH_2_PO_4_/KOH at pH 7.4 in pure D_2_0, containing NaN_3_ and TSP, and centrifuged at 13,400 g and 4°C for 5 min, before 500 μL were transferred to 5 mm tubes.

One-dimensional ^1^H nuclear magnetic resonance (NMR) spectra with water presaturation were recorded for all samples on a Bruker AV 600 spectrometer (Bruker, Fällanden, Switzerland) as previously described [15].

### Spectral Processing and Analysis

#### Spectral processing

All spectra were preprocessed with an in-house program written in GNU Octave [11], which first performed a zero-order phase correction on the TSP signal and then subtracted separate linear baselines from the regions up- and downfield from the water artifact. Finally, the spectral axes of the plasma samples were referenced to the glucose doublet at 5.23 parts per million (ppm) relative to the operating frequency of the spectrometer, and the urine samples to the TSP signal at 0.0 ppm. All spectra were clipped to the spectral range between -0.5 and 9.0 ppm. Further processing and analysis was carried out with the statistics environment R [16]. The spectra were normalized to the area under the TSP signal. In plasma, this signal showed a varying degree of broadening speculated to be due to interaction with macromolecules such as lipoproteins [17,18]. As a quantitative estimate of this effect, the peak width of the TSP signal was determined, anticipating that wider peaks correspond to higher lipoprotein content in the sample. An adaptive, nonlinear baseline was then subtracted using the Barkauskas-Xi-Rocke (BXR) algorithm implemented in the R package “FTICRMS” [19,20]. Especially in the case of plasma, this procedure removed the lipid and protein background and made multivariate analysis of the smaller metabolites possible. The water artifact, the TSP signal and (for urine) the urea region of the spectra were subsequently deleted.

#### Relations between the spectra and endpoints

The urine and plasma spectra, respectively, were scaled to unit variance and subjected to principal component analysis (PCA, using the R package “pcaMethods” [21]) to observe overall variations between the samples. To investigate the relations between the spectra and various endpoints, partial least-squares analysis (PLS, using the R package “pls” [22]) with segment-wise cross validation was employed: Discriminant analysis (PLS-DA) was performed between spectra from different intervention groups or time points, and PLS regression between spectra and continuous endpoints. The goodness of fit and cross-validation reliability was estimated by the diagnostic parameters R^2^ and Q^2^ [23]. In addition, the number of misclassifications (NMC) of the PLS-DA models was also calculated, based on 500 repetitions of the cross validation, and compared to the distribution of classifications with randomly permutated class labels [24]. The loading weights of the PLS models were reverted from unit variance to natural scaling and visualized as interpretable spectra, color-coded to the calculated loading weights [25].

Known identifiable metabolites were quantified by comparing the area under their spectral signals with that of the TSP reference. A number of consistent but unidentified signals were also measured, but their concentrations are consequently only reported in arbitrary units due to the unknown numbers of contributing protons per signal.

#### Statistical analyses

Metabolite concentrations were log-transformed and used for analysis of variance (ANOVA) and subsequent pairwise t-tests with respect to the classification of intervention groups. The metabolite concentrations at the different time points are presented as (back-transformed) medians and interquartile ranges. Change over time was assessed using pairwise t-tests on the metabolites’ log differences and presented as fold change (FC). Pearson correlations were calculated between time to asystole or time to ROSC and the log-transformed metabolite concentrations at all time points. Concentrations at baseline (t1) were compared between surviving and non-surviving piglets using unpaired t-tests.

The pairwise FC rates and t-test p-values derived from the plasma profiles at t1 and t2, i.e. the changes between baseline and 2 h after ROSC, were used as input in an Ingenuity IPA Core Analysis (Build version: 131235, Content version: 11904312; Ingenuity Systems, Inc., Redwood City, CA, USA), mapping the observed changes onto known pathways, functions and diseases.

To further investigate the relationship between the plasma and urine samples, and between metabolites within the samples, Pearson correlations were calculated for all pairs of log-transformed concentration variables at all sampling time points. The correlations were presented as a clustered matrix with rows and columns arranged such that correlated variables are close to each other. This analysis was also repeated separately for the three time points. However, in these matrices the clustering was performed independently for the plasma and urine variables. Additionally, the lower right half of the (symmetric) matrices was filtered to only show strong correlations where the absolute value of r was >0.5.

## Results

### Impact of Mode of Resuscitation

#### Plasma

PLS-DA classification of plasma NMR spectra with respect to the different resuscitation protocols yielded almost entirely insignificant models at baseline (t1) and 2 h (t2) and 4 h (t3) after ROSC. Only the group resuscitated with a C:V ratio of 15:2 (group 6) produced positive Q^2^ values, indicating a weak association between resuscitation mode and the metabolite profile, but no classification was possible at either time point. This was supported by ANOVA of the log-transformed concentrations, where none of the measured compounds, known or unknown, showed significant differences between the resuscitation groups.

#### Urine

The urine spectra invariably produced insignificant PLS-DA models with respect to the resuscitation groups. No Q^2^ values were positive, i.e. the cross-validated classification results were not correlated with the resuscitation protocols, and none of the log-transformed concentrations became significantly different in ANOVA at any of the time points both for TSP and creatinine-normalized spectra.

Since there were no consistent differences between the metabolite profiles from the six different resuscitation groups, they were pooled and considered as equal in the following analyses in order to explore the association between the metabolite profile and the severity of asphyxia and outcome, irrespective of resuscitation protocol.

### Time to ROSC, Duration of Hypoxia, Survival

Separate PLS regression at the three time points found no spectral features of either the plasma or the urine samples to be associated with the response to CPR in terms of the time to ROSC in seconds.

There was a weak Pearson correlation between plasma sample constituents at baseline (t1) and the subsequent duration of hypoxia needed to induce cardiac arrest. The lower the levels of hypoxanthine (r = -0.23, p < 0.01), the longer the piglet would endure before suffering asystole. There was also an effect of the width of the TSP signal, as a broader TSP peak was correlated with a shorter duration of hypoxia (r = 0.26, p < 0.004). These findings were not recapitulated in the urine samples.

Finally, neither plasma nor urine sample constituents at baseline could predict the intervention outcome, as no identifiable markers differed between survivors and non-survivors.

### Metabolite Profile during Hypoxia and Recovery

#### Plasma

**Fig 1:**
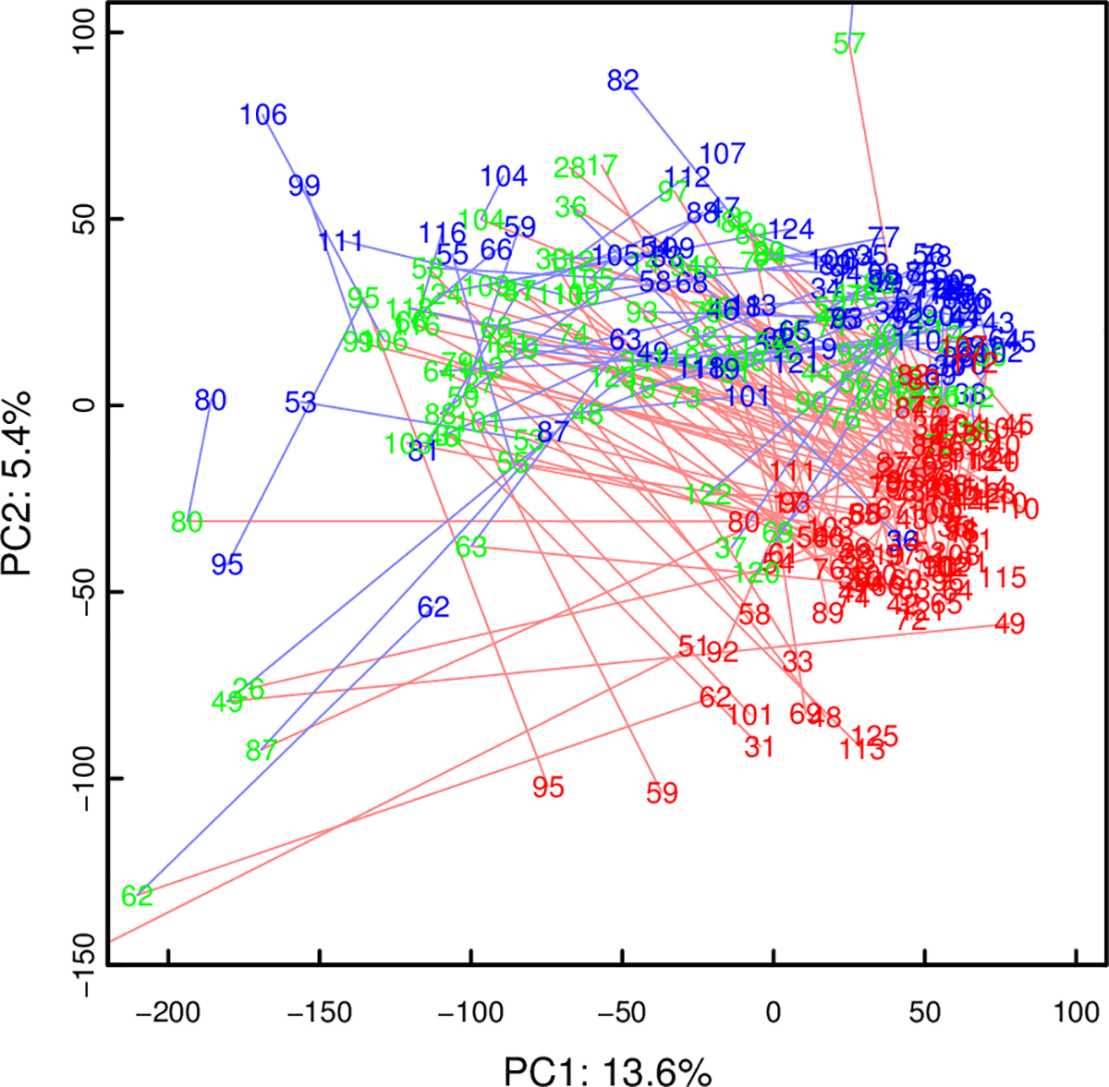
PCA scores plot of plasma spectra. Time points t1, t2, and t3 are red, green, and blue, respectively. Red lines connect time points t1 and t2 of the same animal, blue lines time points t2 and t3. Note how spectra from time point t1, and to a lesser degree time point t3, are clustered while those from time point t2 show more variation.

There was no difference in plasma spectra between groups of different resuscitation approaches. All samples at baseline (t1) and after ROSC at 2 h (t2) and 4 h (t3) were therefore pooled. PCA of the plasma spectra (Fig 1) showed the samples from baseline (t1) to be clearly clustered, and to progress more or less uniformly towards time point t2. The t2 samples exhibited much more variability, and overlapped strongly with t3 samples, although a trend seemed to be apparent. The cluster center at t3 was not equal to baseline (t1).

**Fig 2:**
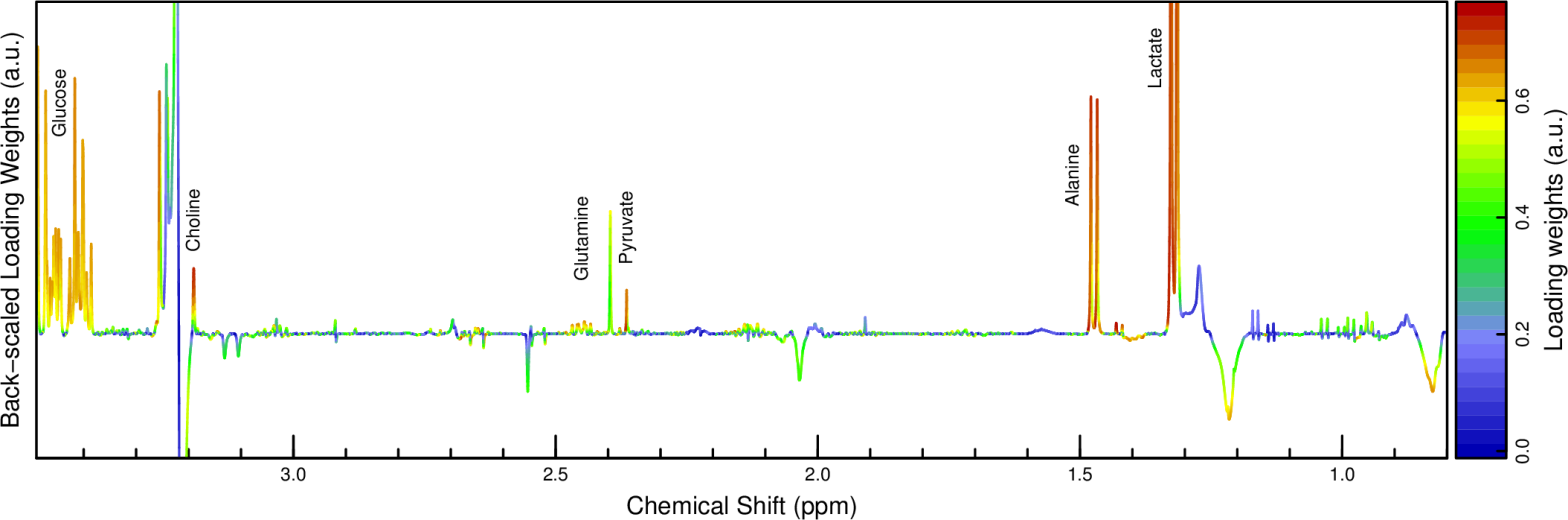
Backscaled PLS-DA loading weights for progression of plasma profiles from time point t1 to t2. First PLS component. The amplitude corresponds to covariance, the color scale to correlation with the group discrimination. The loading weights indicate a consistent increase in among others lactate, alanine and glucose while lipid signals seem to decrease.

**Fig 3:**
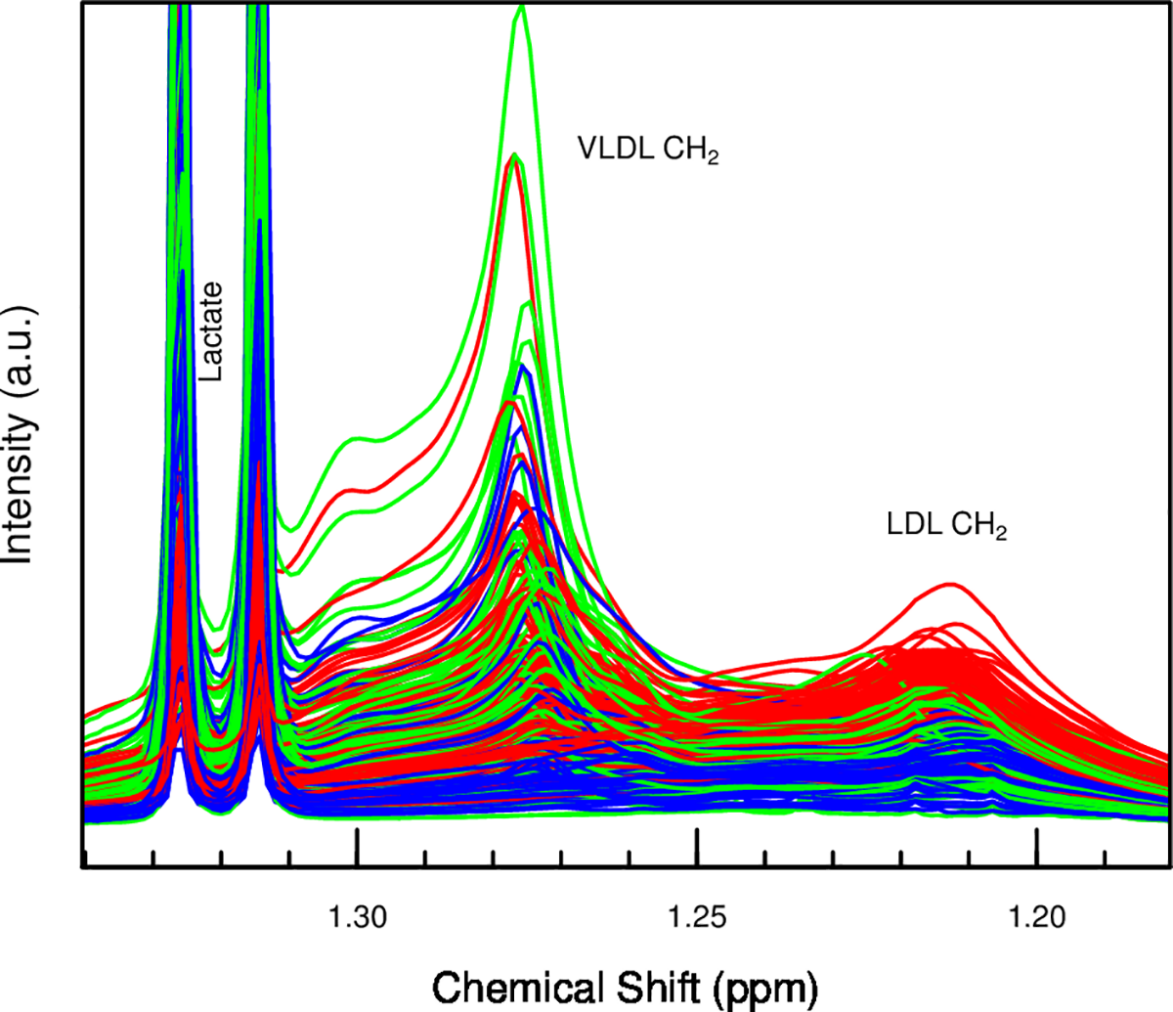
Lipid region of plasma spectra. Color-coded red, green, blue for time points t1, t2, t3, before subtraction of baseline. Note the clear decrease in the LDL associated signal.

PLS-DA of plasma spectra between t1 and t2 resulted in a model that was predictive (R^2^ of 66%) and valid (7-fold segment-wise CV: Q^2^ of 64%, one component). The median NMC of 22 (95% CI: 22–24) was significantly lower than that of permutated class labels (107 (93–128), p < 5e-20). A loading plot (Fig 2) indicated a strong and consistent increase of plasma concentrations of lactate, alanine, pyruvate, succinate, glucose, hypoxanthine, branched-chain amino acids (BCAA) and some undefined metabolites, and a decrease of NMR signals associated with lipids. Additionally, the peak positions of EDTA and citrate moved slightly to the left, i.e. higher frequencies, indicating a decrease in pH. The decrease of the lipids signals was also assessed visually by plotting the plasma spectra together (Fig 3).

PLS-DA of plasma spectra between t2 and t3 did not reveal a significant difference between the spectra (R^2^ of 28%, Q^2^ of 20%; NMC equal for real and permutated labels, p = 0.92).

Lastly, the comparison of plasma spectra at time points t3 vs. t1 with PLS-DA yielded a significant model (R^2^ of 76%, Q^2^ of 72% with one component) and the median NMC of 13 (12–16) was substantially smaller than random NMC of 97 (82–114), p < 2e-23, indicating that the metabolic profiles had not returned to baseline 4 h after ROSC. At t3, NMR signals associated with lipids were weaker and choline stronger than at baseline. Lactate and alanine concentrations were also higher, but much more ambiguous than at t1 and t2. A univariate characterization of a selection of metabolite concentrations, along with their changes between the time points, is presented in Table 3.

**Table 3:** Concentrations of plasma metabolites at different time points, and individual, pairwise fold-change between them.

Compound	ppm	Median concentrations (IQR) in mM			Pairwise FC (p- and q-value)	
		t1	t2	t3	t1 to t2	t2 to t3
Acetate	1.91	0.0053 (0.000061–0.018)	0.014 (0.00023–0.087)	0.014 (0.0028–0.1)	2.82 (1.5e-09; 2.67e-09)	1.6 (0.0033; 0.0056)
Alanine	1.48	0.64 (0.37–1.0)	1.5 (0.64–2.6)	1.2 (0.43–2.6)	2.21 (4.4e-37; 7.04e-36)	-1.38 (2.5e-11; 1.6e-10)
Choline	3.19	0.013 (0–0.049)	0.061 (0.012–0.14)	0.077 (0.021–0.22)	4.85 (1.8e-26; 5.24e-26)	1.22 (5.7e-05; 1.3e-04)
Creatinine	4.05	0.034 (0.023–0.05)	0.045 (0.022–0.063)	0.061 (0.038–0.082)	1.29 (8.4e-15; 1.68e-14)	1.27 (5.7e-14; 9.12e-13)
DMA	2.72	0.0078 (0.0042–0.012)	0.0088 (0.0048–0.012)	0.0099 (0.0058–0.013)	1.15 (4.2e-05; 5.84e-05)	1.09 (0.011; 0.17)
Formate	8.45	0.0083 (0–0.043)	0.012 (0–0.054)	0.018 (0–0.035)	1.4 (0.078; 0.86)	1.81 (0.00019; 0.00038)
Fumarate	6.51	0.0056 (0.0017–0.013)	0.033 (0.0047–0.083)	0.019 (0.0012–0.085)	4.85 (3.9e-33; 3.12e-32)	-1.56 (1.1e-06; 3.2e-06)
Glucose	5.24	6 (4.1–8.6)	11 (5.5–20)	6.6 (2.7–16)	1.77 (4.3e-32; 2.29e-31)	-1.53 (8.8e-14; 9.39e-13)
Glutamine	2.45	0.27 (0.19–0.4)	0.45 (0.25–0.76)	0.51 (0.25–1.0)	1.65 (1.9e-32; 1.22e-31)	1.06 (0.055; 0.074)
Glycerol	3.57	0.18 (0.0041–0.39)	0.35 (0.045–1.0)	0.2 (0.018–2.2)	1.8 (3.5e-06; 5.09e-06)	-1.2 (0.06; 0.08)
Glycine	3.55	0.61 (0.39–1.0)	0.95 (0.39–1.5)	0.89 (0.43–1.7)	1.44 (1.3e-23; 3.2e-23)	1.03 (0.56; 0.60)
Hypoxanthine	8.20	0.041 (0.023–0.068)	0.12 (0.036–0.26)	0.06 (0.017–0.27)	2.63 (1.0e-27; 3.2e-27)	-1.6 (3.8e-09; 1.52e-08)
Isoleucine	1.00	0.097 (0.061–0.16)	0.1 (0.055–0.2)	0.065 (0.044–0.18)	1.06 (0.15; 0.16)	-1.39 (3.5e-11; 1.9e-10)
Lactate	1.33	2.9 (1.5–5.3)	10 (3.7–16)	5.3 (1.3–16)	3.07 (2.1e-38; 6.72e-37)	-1.75 (2.1e-16; 6.7e-15)
Leucine	0.95	0.11 (0.074–0.17)	0.14 (0.054–0.32)	0.089 (0.053–0.29)	1.23 (0.00024; 0.00032)	-1.37 (5e-10; 2.3e-9)
Malate	2.65	0.013 (0–0.062)	0.13 (0.013–0.28)	0.088 (0.02–0.29)	6.69 (3.0e-20; 6.4e-20)	-1.07 (0.35; 0.40)
Methionine	2.63	0.032 (0.0019–0.077)	0.032 (0.0045–0.087)	0.03 (0–0.088)	1.02 (0.79; 0.79)	-1.22 (0.05; 0.07)
Myo-inositol	4.06	0.064 (0–0.39)	0.2 (0.045–0.6)	0.23 (0.037–0.6)	2.26 (1.6e-12; 3.01e-12)	1.02 (0.71; 0.71)
Phenylalanine	7.33	0.051 (0.026–0.079)	0.064 (0.018–0.14)	0.046 (0.018–0.13)	1.22 (9e-04; 1.15e-03)	-1.27 (1.0e-06; 3.2e-06)
Proline	4.14	0.49 (0.32–0.84)	0.57 (0.34–0.92)	0.57 (0.32–0.94)	1.08 (0.0022; 0.0027)	-1.09 (0.00075; 0.0014)
Pyruvate	2.37	0.1 (0.059–0.21)	0.21 (0.11–0.34)	0.2 (0.058–0.33)	2.0 (1.5e-29; 5.33e-29)	-1.22 (0.00083; 0.0015)
Succinate	2.40	0.015 (0.0054–0.035)	0.17 (0.0095–0.62)	0.072 (0.0035–0.44)	7.67 (1.2e-30; 5.49e-30)	-1.54 (4.7e-05; 1.16e-04)
Trimethylamine	2.88	0.0011 (0–0.0033)	0.0046 (0.00083–0.016)	0.0054 (0.00092–0.019)	4.43 (4.7e-34; 5.01e-33)	1.26 (0.0097; 0.016)
Tyrosine	7.20	0.11 (0.059–0.2)	0.13 (0.066–0.22)	0.11 (0.047–0.19)	1.21 (1.6e-06; 2.44e-06)	-1.22 (6.1e-09; 2.17e-08)
Valine	1.03	0.19 (0.11–0.34)	0.22 (0.082–0.45)	0.16 (0.069–0.36)	1.08 (0.12; 0.13)	-1.36 (1.4e-11; 1.12e-10)
Unknown doublet	1.06	1.6 (0.094–4.5)	2.1 (0.0015–5.1)	1.9 (0.7–4.6)	1.34 (0.012; 0.015)	-1.04 (0.66; 0.68)
Unknown doublet	1.11	2.7 (1.8–3.8)	2.2 (0.33–3.7)	2.4 (1.1–3.9)	-1.59 (2.1e-07; 3.36e-07)	1.09 (0.22; 0.27)
Unknown doublet	1.14	0.19 (0.12–0.47)	0.14 (0.083–0.43)	0.14 (0.063–0.28)	-1.27 (1.4e-08; 2.36e-08)	-1.07 (0.034; 0.05)
Unknown multiplet	1.40	40 (24–63)	23 (9.1–43)	25 (9.4–43)	-1.92 (1.2e-29; 4.8e-29)	-1.06 (0.46; 0.51)
Unknown singlet	3.92	2.7 (1.3–7.7)	3.3 (0.8–14)	3.4 (1.4–25)	1.14 (0.025; 0.029)	1.3 (0.00013; 0.00028)
Unknown singlet	5.39	1.1 (0.24–2.8)	1.9 (0.25–6)	2.6 (0.34–8)	1.78 (6.6e-23; 1.51e-22)	1.16 (8e-06; 2.2e-05)
*FWHM of TSP*		0.0047 (0.003–0.007)	0.004 (0.0026–0.0052)	0.0038 (0.0027–0.0051)	-1.24 (6.1e-25; 1.63e-24)	-1.02 (0.26; 0.31)

Time point t1: baseline, between surgical preparation and asphyxiation; t2: 2 hours of rest after resuscitation; t3: 4 hours of rest. Concentrations are reported in mM, except for the unidentified signals; their values are in arbitrary units proportional to actual concentrations. FWHM (full width at half maximum) of the TSP signal is affected by plasma lipoproteins and is reported in spectral ppm.

#### Urine

**Fig 4:**
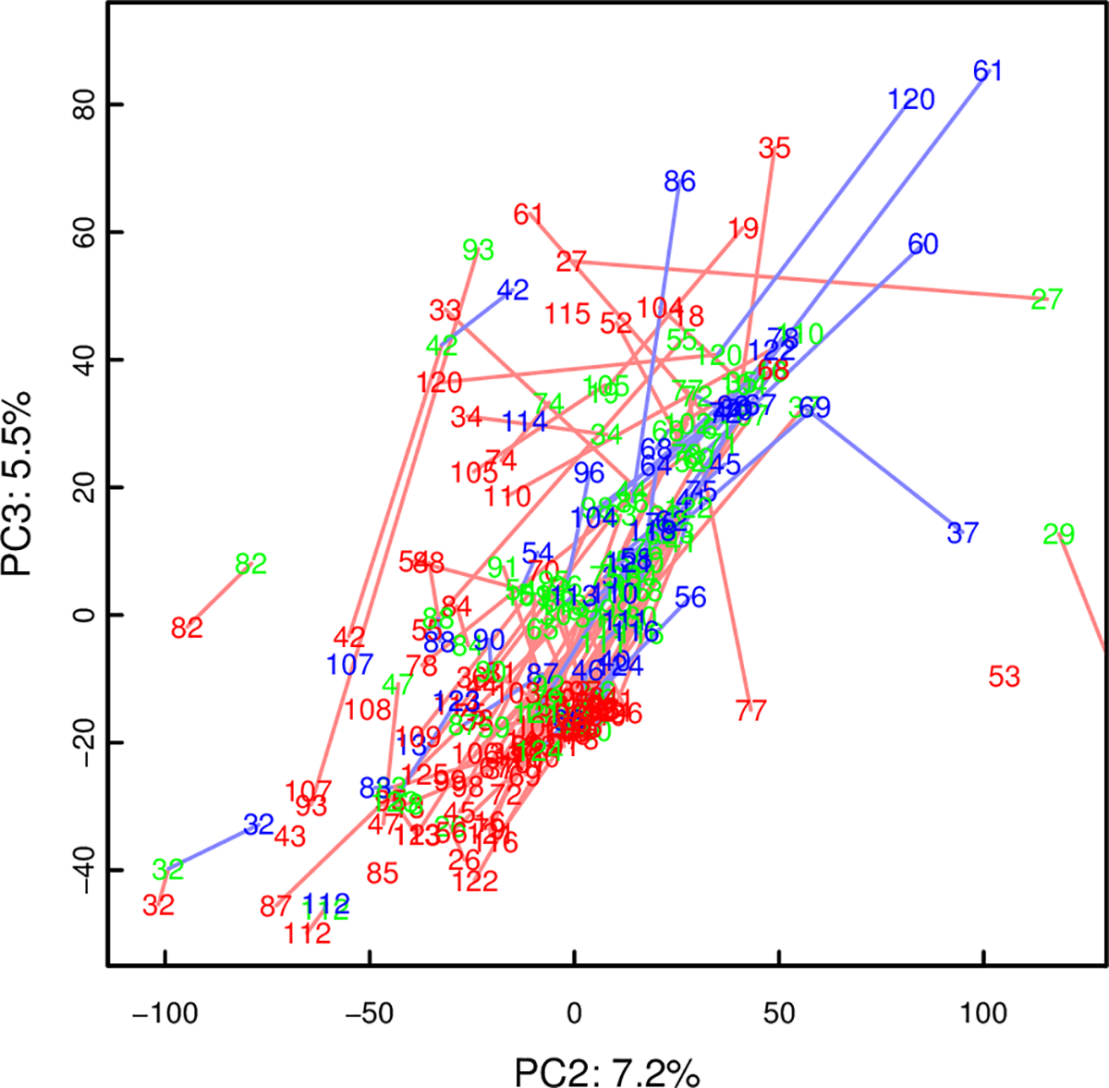
PCA scores plot of urine spectra. UV-scaled, same color-code as plasma PCA above. Showing components 2 and 3 where the separation between the time points was more apparent.

There was no difference in urine spectra between groups of different resuscitation protocols. All samples at the three time points were therefore pooled. PCA (Fig 4) and PLS-DA of the urine spectra showed much less apparent separation between the time points than the plasma samples above. The model between t1 and t2 managed to predict reasonably well (R^2^ 37%, Q^2^ 28%; median NMC 38 (35–40), and better than random NMC of 89 (74–108), p < 2e-9), based on changes in urine lactate, alanine, BCAA, glucose, and hypoxanthine concentrations.

As with plasma, there was no significant difference between t2 and t3 by PLS-DA (R^2^ of 18%, Q^2^ 0%; NMC not different from random, p = 0.96), while the comparison of t3 and t1 yielded an even better model (R^2^ 43%, Q^2^ 37%; median NMC 28 (25–30) compared with random NMC 52 (45–62), p < 6e-8) than baseline vs. t2 above. The BCAA, especially valine, but also lactate, alanine, glucose, and choline concentrations in urine were clearly higher than at baseline. Selected urinary metabolite concentrations are shown in Table 4.

**Table 4:** Concentrations of urine metabolites at different time points, and individual, pairwise fold-change between them.

Compound	ppm	Median concentrations (IQR) in mM			Pairwise FC (p- and q-value)	
		t1	t2	t3	t1 to t2	t2 to t3
1-Methylnicotinamide	8.95	0.14 (0.042–0.93)	0.15 (0.055–0.7)	0.19 (0.055–0.6)	1.14 (0.077; 0.087)	1.16 (0.017; 0.05)
3-Hydroxyisovalerate	1.27	0.035 (0.025–0.078)	0.03 (0.022–0.068)	0.028 (0.02–0.06)	-1.18 (1.4e-09; 4.08e-09)	-1.1 (4.2e-08; 1.5e-06)
Alanine	1.48	0.42 (0.17–1.5)	1.1 (0.4–2.9)	1.3 (0.54–4.5)	2.29 (1.7e-18; 1.49e-17)	1.25 (0.00025; 0.0022)
Ascorbate	4.52	0.83 (0.11–3.9)	0.9 (0.17–3.4)	0.86 (0.15–3.2)	1.21 (0.059; 0.069)	-1.04 (0.66; 0.75)
Choline	3.21	0.12 (0.041–0.67)	0.29 (0.051–1.1)	0.32 (0.076–1.9)	1.91 (1.1e-10; 4.28e-10)	1.12 (0.084; 0.16)
Creatinine	4.05	3 (1.5–8.6)	2.5 (0.92–7.4)	2 (0.76–5.1)	-1.27 (0.001; 0.0014)	-1.17 (0.02; 0.05)
Formate	8.47	0.49 (0.16–1.2)	0.31 (0.12–0.92)	0.26 (0.11–0.86)	-1.32 (0.0068; 0.0092)	-1.25 (0.00057; 0.003)
Fumarate	6.52	0.22 (0.077–1.2)	0.29 (0.11–0.98)	0.25 (0.1–0.55)	1.24 (0.00071; 0.0011)	-1.09 (0.021; 0.05)
Glucose	5.24	2 (0.5–7.9)	10 (2.3–50)	13 (2.3–70)	5.2 (1.3e-20; 1.5e-19)	1.2 (0.023; 0.051)
Glycine	3.57	2.4 (0.56–9.8)	2.8 (1–7.2)	2.9 (0.99–11)	1.39 (1.0e-05; 2.5e-05)	1.04 (0.43; 0.54)
Hippurate	7.55	0.71 (0.099–5.4)	0.75 (0.18–4.3)	0.57 (0.067–4)	1.28 (0.043; 0.054)	-1.19 (0.14; 0.20)
Hypoxanthine	8.21	0.13 (0–0.79)	0.41 (0.065–1.4)	0.38 (0.07–1.7)	3.44 (1.4e-12; 7e-12)	1.1 (0.10; 0.17)
Lactate	1.33	1.2 (0.81–3.7)	9.7 (1.6–28)	12 (1.8–32)	6.17 (8.8e-28; 3.08e-26)	1.14 (0.087; 0.15)
Leucine	0.97	0.058 (0.039–0.1)	0.089 (0.051–0.26)	0.1 (0.053–0.21)	1.59 (3.4e-11; 1.49e-10)	1.1 (0.0099; 0.04)
Lysine	1.72	0.14 (0–2.3)	0.16 (0–0.89)	0.16 (0.035–0.89)	-1.11 (0.37; 0.41)	1.04 (0.71; 0.75)
N,N-Dimethylglycine	2.93	1 (0.21–3.1)	0.63 (0.16–2.4)	0.53 (0.071–2)	-1.45 (1.9e-06; 5.12e-06)	-1.23 (0.0062; 0.027)
N-phenylacetylglycine	7.43	1.3 (0.45–6.8)	0.92 (0.2–4.7)	0.84 (0.18–3.3)	-1.38 (0.00021; 0.00037)	-1.17 (0.10; 0.17)
Succinate	2.41	0.28 (0.088–1)	0.6 (0.18–1.3)	0.48 (0.17–1.2)	1.92 (2.2e-15; 1.28e-14)	1 (0.94; 0.94)
Trimethylamine	2.88	0.15 (0–1.2)	0.16 (0–0.72)	0.17 (0–1.5)	1.09 (0.45; 0.48)	1.2 (0.2; 0.3)
Trimethylamine-N-Oxide	3.27	1.5 (0–6)	1.3 (0–4)	1.2 (0–3.8)	-1.03 (0.8; 0.8)	1.01 (0.93; 0.96)
Valine	1.05	0.039 (0.016–0.093)	0.12 (0.03–0.35)	0.14 (0.042–0.35)	2.81 (5e-21; 8.8e-20)	1.42 (0.00012; 0.0014)
Unknown doublet	1.08	0.059 (0.038–0.24)	0.062 (0.042–0.15)	0.062 (0.04–0.21)	-1.02 (0.61; 0.63)	-1.06 (0.0049; 0.025)
Unknown doublet	1.11	0.089 (0.069–0.17)	0.085 (0.067–0.16)	0.081 (0.065–0.12)	-1.06 (0.016; 0.021)	-1.03 (0.039; 0.08)
Unknown doublet	1.15	0.16 (0.05–0.48)	0.3 (0.12–0.74)	0.3 (0.15–0.71)	2.06 (6.5e-17; 4.55e-16)	1.06 (0.10; 0.17)
Unknown doublet	1.25	0.18 (0.11–0.37)	0.12 (0.061–0.29)	0.11 (0.052–0.2)	-1.46 (4.1e-10; 1.44e-09)	-1.19 (5.6e-05; 9.8e-04)
Unknown doublet	1.38	0.069 (0.033–0.21)	0.062 (0.029–0.19)	0.057 (0.026–0.15)	-1.16 (0.00089; 0.0013)	-1.02 (0.58; 0.68)
Unknown doublet	5.1	0.0038 (0–0.0057)	0.0059 (0.0015–0.022)	0.007 (0.0027–0.03)	1.99 (9.1e-10; 2.9e-09)	1.16 (0.016; 0.051)
Unknown doublet	5.48	0.00074 (0–0.0054)	0.00056 (0–0.0038)	0.00062 (0.0002–0.0031)	-1.31 (9.1e-05; 1.8e-04)	-1.08 (0.32; 0.42)
Unknown doublet	5.7	0.12 (0.019–0.54)	0.098 (0.0061–0.45)	0.082 (0.013–0.39)	-1.38 (7.8e-05; 1.6e-04)	-1.16 (0.079; 0.15)
Unknown multiplet	6.65	0.0072 (0.0025–0.028)	0.0044 (0.0014–0.028)	0.0042 (0–0.015)	-1.42 (0.00017; 0.00032)	-1.17 (0.23; 0.31)
Unknown multiplet	6.76	0.011 (0.0054–0.037)	0.0081 (0.003–0.034)	0.008 (0.0012–0.018)	-1.33 (0.00051; 0.00081)	-1.26 (0.017; 0.05)
Unknown multiplet	6.79	0.0081 (0.0034–0.027)	0.0057 (0.0011–0.026)	0.0041 (0.00045–0.014)	-1.53 (4.9e-05; 1.1e-04)	-1.45 (0.0034; 0.02)
Unknown multiplet	7.68	0.18 (0.083–0.53)	0.14 (0.047–0.46)	0.12 (0.029–0.29)	-1.32 (0.00044; 0.00074)	-1.24 (0.015; 0.053)
Unknown singlet	6.78	0.0034 (0–0.017)	0.0016 (0–0.011)	0.0013 (0–0.0095)	-2.12 (1.6e-05; 3.7e-05)	-1.06 (0.69; 0.76)
Unknown triplet	6.29	0.0062 (0–0.067)	0.003 (0–0.039)	0.0032 (0–0.031)	-1.4 (0.05; 0.06)	-1.1 (0.5; 0.6)

Time point t1: baseline, between surgical preparation and asphyxiation; t2: 2 hours of rest after resuscitation; t3: 4 hours of rest. Concentrations are reported in mM, except for the unidentified signals; their values are in arbitrary units proportional to actual concentrations.

#### Metabolic pathways

The concentration FC between t1 and t2, i.e. the response to the hypoxia and resuscitation, of the metabolites in both plasma and urine was analyzed with the pathway mapping tool Ingenuity. In both urine and plasma, Ingenuity indicated that the metabolite changes from t1 to t2 were associated with gastrointestinal and hepatic disorders and inflammatory reactions. The observed metabolite patterns were linked to networks annotated with cardiac damage, infarction and heart failure, liver damage and necrosis, and with renal failure.

### Correlation of urine and plasma metabolites

**Fig 5:**
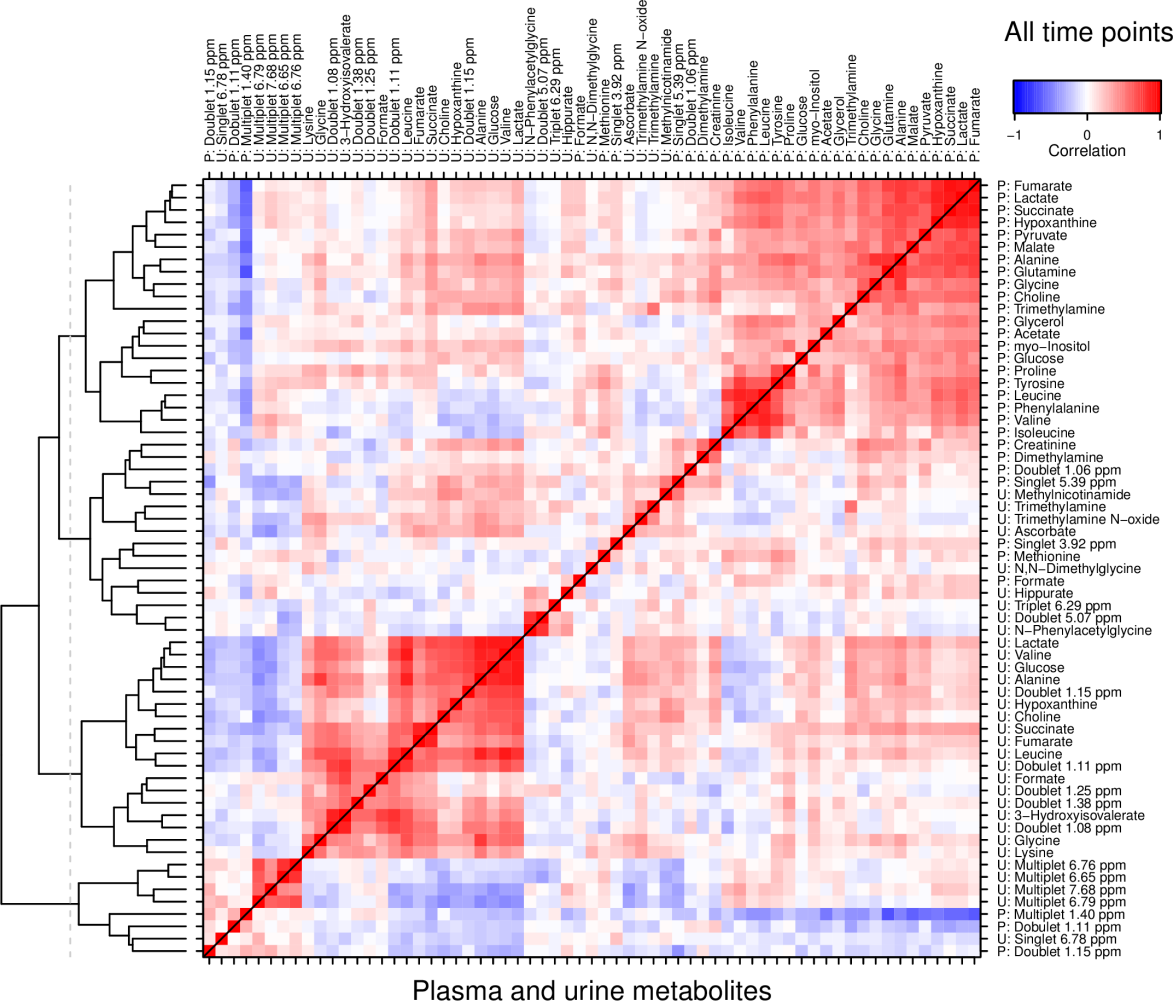
Correlations between plasma and creatinine-normalized urine metabolite concentrations at all time points combined. Left margin shows dendrogram from hierarchical cluster analysis (HCA) by which rows and columns are ordered. Two major, separate clusters emerge for plasma (top right) and urine metabolites (bottom left), suggesting a low overall coherence between the two, and justifying treating the two independently in further analysis.

The correlation matrix derived from the log-transformed concentration variables of matching pairs of plasma and creatinine-normalized urine samples is shown in Fig 5. Two large clusters of correlated metabolites emerged, which were homogenous in terms of sample material: One cluster contained only plasma, the other only urine variables. Outside the two main clusters remained largely urine variables, interspersed with very few of the plasma variables. Note also that the inter-material correlations were much weaker than those in the respective plasma and urine clusters.

Considering only t1 samples (Fig A in S1 Supplement), the plasma concentrations of lactate, hypoxanthine, fumarate and succinate were already correlated, as were a number of amino acids. In urine, a larger cluster implies similar, but somewhat stronger correlations. As in the full dataset, there was little coherence between plasma and urine metabolite concentrations.

**Fig 6:**
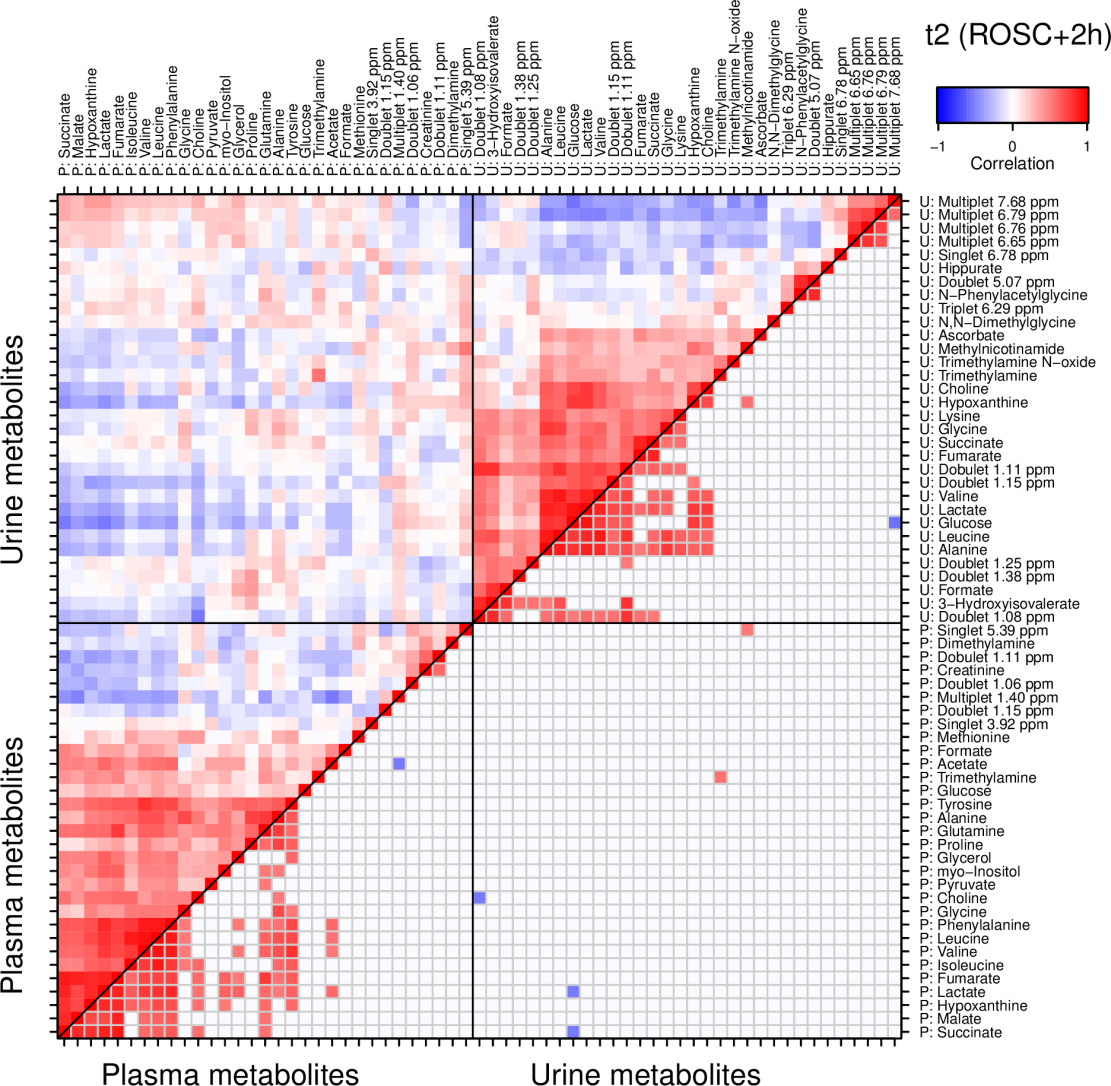
Correlations between plasma and creatinine-normalized urine metabolite concentrations at time point t2. 2 hours after return of spontaneous circulation (ROSC). The plasma (lower left) and urine (upper right) submatrices are ordered by independent hierarchical clustering (HCA). The lower right half of the entire matrix (i.e. below the diagonal) is truncated to only show strong correlations with |*r*|>0.5. Note again that while there are groups of correlated metabolites within the plasma and urine subsets, the correlation between the materials is comparably weak.

At t2 (Fig 6), the plasma and urine profiles were dominated by the concerted increase of many metabolites described above, producing large clusters of interrelated variables. The correlation between plasma and urine, on the other hand, was of an inverse nature, and overall weak. This tendency continued at t3 (Fig B in S1 Supplement). The correlations within the sample materials were strong, while those between the materials were weaker and largely inverse.

**Fig 7:**
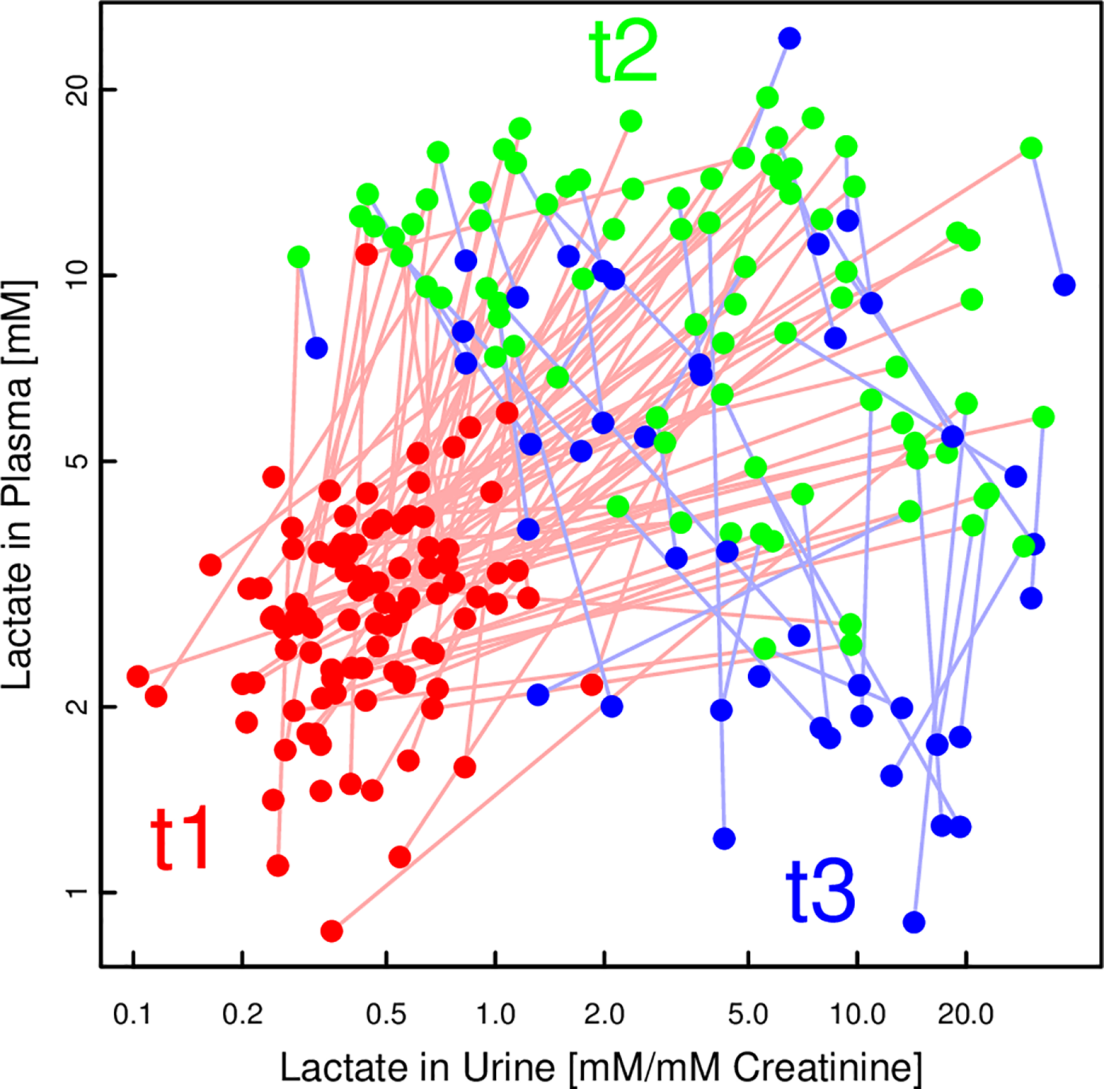
Scatter plot of lactate concentrations of individual pigs at all time points, in plasma and urine. While both plasma and urine lactate concentrations have increased between t1 (before asphyxiation; red) and t2 (2h after ROSC; green), the subsequent decrease in plasma at t3 (4h after ROSC; blue) is not yet reflected in urine.

Tracing the lactate concentrations over time (Fig 7) revealed a lag between plasma and urine: While plasma lactate levels increased from t1 to t2 and then decreased towards t3, urinary lactate levels stayed high at t3, or even increased slightly more. A similar pattern was also observed for the concentrations of other metabolites, in particular the BCAA (see Tables 3 and 4 and the cross-correlation in Fig 5).

## Discussion

The present study demonstrates the severe metabolomic changes in plasma and urine that occur during asphyxiation and subsequent resuscitation of newborn pigs. The metabolic profiles of both plasma and urine were broadly altered between baseline and 2 h after ROSC. While the plasma profiles began to normalize at 4 h after ROSC, urine showed no such trend. Neither of them returned to their baseline state, which reflects the severity of the impact that the experiment had on the animals. Skappak *et al*. [26] recently investigated whether a hypoxic insult can be diagnosed by urinary NMR analysis, and also found that the metabolite profiles were significantly affected after 4 h of recovery.

This is the first NMR study of metabolic profiles after cardiac arrest with cardiopulmonary resuscitation (as opposed to ‘re-oxygenation’ in other asphyxia studies). In this model we previously examined differences with clinical and more established biochemical methods including Enzyme-linked immunosorbent assay (ELISA) and Real-Time Polymerase Chain Reaction (RT-PCR) and found few differences between resuscitation protocols [27,28]. We speculated that the experimental protocol in itself generated such extensive tissue damage and inflammation that the relative contribution of the different resuscitation protocols would not be measurable. We wanted to investigate this at the level of the metabolism using NMR. However, in accordance with our previous work [2–5], we found no evidence for a differential metabolic response to the different resuscitation protocols. NMR may therefore not be more advantageous than ELISA and RT-PCR in comparison of different experimental protocols of asphyxia and resuscitation. However, the method is potentially useful in delineating the question of individual susceptibility to hypoxia, as well as to describe the metabolic pathways and mechanisms involved in perinatal asphyxia.

A profiling study of less asphyxiated pigs by Solberg *et*. *al*. [7] also found a generally similar impact of different modes of reoxygenation on the plasma metabolites, but observed a faster reduction of the increased succinate, fumarate and alpha-ketoglutarate concentrations when using air instead of higher oxygen concentrations. Also for urine profiles, a study by Fanos *et al*. [9] found differences between different reoxygenation protocols. While we could not confirm these inspiring results, the metabolic changes they report between baseline and after hypoxia and recovery match ours closely.

Independently of resuscitation protocol, we observed a 7.7- and 4.9-fold increase in plasma succinate and fumarate concentrations, respectively, 2 h after ROSC (t2) compared with baseline (t1), whereas the changes in urine at the same time points were more modest and only 1.9 and 1.2-fold. The urine concentrations of the metabolites 4 h after ROSC (t3) were almost back to baseline, and almost in parallel with the reduction of plasma concentrations. The near normalization of urine succinate and fumarate at t3 is in contrast to the concentration changes of other metabolites such as lactate and hypoxanthine, which increased markedly at t2 followed by sustained high urine concentrations at t3, in contrast to the fall in plasma concentrations. Some of the differences can be explained by differences in renal handling of the metabolites. Succinate is actively reabsorbed in the proximal tubules by an apical dicarboxylate transporter, which is activated by lowering extracellular pH as in asphyxia [29]. Lactate reabsorption in the proximal tubules is passive and driven by a transtubular concentration gradient. Studies of rats have shown that the gradient is generated by the consumption of lactate for gluconeogenesis by the renal cortex [30]. In normoxemic sheep, lactate metabolism accounts for 43% of renal O_2_ consumption [31]. During acute hypoxemia the renal metabolism has been shown to be altered from net glucose release and net lactate uptake to net glucose uptake and net lactate release. Urine metabolic biomarkers of hypoxemia have to take into account the differences in renal handling of the metabolites. For future studies of different resuscitation protocols the number of sampling time points, especially immediately after hypoxia, will be important for the evaluation of metabolic effects.

A study by Atzori *et al*. [8] reported multivariate associations between urine profiles of asphyxiated pigs and worst vs. best outcome, i.e. death vs. particularly short recovery time. In our material, despite including severely compromised animals, it was not possible to correlate individual variations in the recovery time, i.e. the time to ROSC, to markers or patterns in any biofluid profiles before or after the asphyxia.

Solberg *et al*. [7] reported a correlation between plasma metabolites at baseline and the individual pig’s susceptibility to hypoxia. They identified a panel of metabolites with concentrations and concentration ratios that predicted the duration of hypoxia until resuscitation had to be initiated due to hypotension or moderate metabolic acidosis. We were in general unable to confirm the same findings in our material when resuscitation was started at asystole and severe metabolic acidosis of BE<-30 and pH around 6.8. Our samples do nonetheless suggest a weak link between the baseline plasma profile and the subsequent duration of hypoxia: Lower lipoprotein levels indicated by the narrower TSP signal seem to be signs of more resilient pigs. As all baseline plasma samples were drawn after the strict surgical preparation, one could speculate that this metabolic pattern resembles a genuine difference between individual pigs.

In terms of the general characterization of the metabolic trajectories, our plasma data show an increase and subsequent decrease of many profiled substances, most notably lactate, succinate, fumarate, malate and alanine, which could be useful in diagnosis and estimation of prognosis following asphyxia. Choline increased without a subsequent decrease, while the low-density lipoprotein (LDL) lipid signals did the opposite. Choline concentrations in umbilical cord blood was also increased and almost doubled in infants with perinatal asphyxia compared with controls [10].

The role of these metabolites and how they interact in metabolic pathways has not been clarified. Lipids play a role in critical illness, and in particular high-density lipoproteins (HDL) have been shown to have a protective effect in sepsis. [32] Lipid metabolism in hypoxia has been shown to depend on the ambient temperature. [33] During acclimation to 22°C the peripheral lipid uptake in mice was increased and sharply decreased by hypoxia resulting in increased plasma triglyceride and cholesterol levels. At thermoneutrality, plasma lipids was not increased and LDL-cholesterol was decreased by hypoxia. The newborn pigs in this study were kept at thermoneutrality and may be less susceptible to decreased peripheral lipid uptake during hypoxia. The decrease in LDL cholesterol may be explained by a combination of minimal hypoxic reduction of cholesterol uptake, whereas the hepatic output of very low density lipoproteins (VLDL) may have been reduced as suggested in mice by Jun *et al*. [33]

There are, owing to the severe nature of this study, no corresponding studies in humans with which to compare our findings. More generally, though, human studies of newborn asphyxia and hypoxic ischemic encephalopathy (HIE) also found differences in the metabolic profile of healthy compared with asphyxiated newborns, involving many of the substances we have found altered in our study. For example, asphyctic infants were characterized by increased urine lactate and glucose, together with threonine and 3-hydroxyisovalerate, as well as decreased dimethylglycine, dimethylamine, creatine, succinate, formate, urea and aconitate, and succinate [11]. Another study found increased cord blood acetone, 3-hydroxybutyrate, succinate, and glycerol that were altered in severe HIE and predicted HIE severity, [10]. Liquid chromatography coupled with mass spectrometry found metabolites in cord blood that characterized asphyxia, e.g. glycerophospholipids and taurine, whereas metabolites characterizing HIE were alanine, asparagine, isoleucine, methionine, phenylalanine, proline, tyrosine, and valine [34]. Further, in premature neonates NMR identified discriminant metabolites in urine in those who later developed respiratory disease [14], and in neonatal sepsis [13] where non-specific clinical signs, along with the inaccuracy of available biomarkers, sometimes makes a correct risk and severity assessment difficult, NMR could help to uncover features of the disease that are still hidden [12].

The change of the plasma and urinary profiles between the time points before and after asphyxia and resuscitation was analyzed in terms of metabolic pathways and gene networks. The change was found to be consistent with a dramatic reaction of the organism to such a catastrophic event as cardiac arrest and CPR. Not surprisingly, the metabolism switches into an emergency, catabolic state, and there is clear evidence of hypoxemia and acidosis characterized by increased plasma lactate, hypoxanthine and glycerol concentrations, activation of amino acid metabolism and increased plasma creatinine indicating reduced kidney function.

We could directly compare plasma and urine profiles and found them at best loosely correlated. Compared to plasma, the urine metabolite profile represents the final result of glomerular plasma filtration, and tubular reabsorption and excretion during the period from the last emptying of the bladder. The concentration of urine metabolites depends not only on the plasma concentrations of the metabolites, but also on the renal handling of the individual metabolites and sampling frequency. For real-time monitoring of acute conditions such as hypoxia, plasma is superior to urine samples. In fact, one of the important findings of this study may be the poor correlation between plasma and urine samples. This will be useful for those attempting to develop clinical markers for hypoxic injury. Urine samples are delayed and are dependent on renal function. In addition individual metabolites have different clearance patterns. The data support the use of caution when using urine as a marker for metabolite alterations.

The present study has some limitations. The samples are from a study primarily designed to compare the effects of several different resuscitation protocols. There were too few pigs in each group to make reliable comparisons of metabolite profiles between the different protocols. The pigs were so severely asphyxiated in this study, with a heart rate of zero evaluated by electrocardiogram, that more subtle metabolic differences between resuscitation protocols may have been subdued. As there was no difference in outcome between the protocols, metabolite concentrations in plasma and urine during asphyxia and resuscitation were pooled for all resuscitation protocols. The study presents metabolite profiles in plasma and urine at baseline, and 2 h and 4 h after asphyxia, resuscitation and ROSC. It provides a list of metabolites that alone or in combination may be tested as biomarkers of susceptibility to hypoxia, effect of resuscitation protocols and time to ROSC. However, with a limited number of time points we may miss important metabolite changes occurring before 2 h and after 4 h of rest after ROSC.

In this study we present results from NMR spectroscopic analysis which can be validated by mass spectrometry analyses or specific metabolite assays. In Sachse *et al*. [15] both urine creatinine and citrate concentrations were validated by specific enzymatic methods, which were in excellent agreement with NMR spectroscopy measurements (R^2^ > 95%). Other analytical techniques may be used for validation and quantification of biomarkers such as RT-PCR to quantify candidate microRNAs in blood and urine [35]. ELISA and mass spectrometry of cerebrospinal fluid [36] are analytical techniques that have been recently used for studies of perinatal asphyxia. Compared to NMR, RT-PCR, immunochemical analyses and mass spectrometry are more readily available and thus more likely candidates for routine analyses after perinatal asphyxia. NMR may potentially be more advantageous in research by identifying a wider array of known and unknown metabolites. However, as our results imply, NMR may also be useful in measuring individual susceptibility to hypoxic-ischemic insults.

## Conclusion

The study demonstrates the severe metabolic alterations in plasma and urine caused by asphyxia-induced cardiac arrest and CPR. NMR-based profiling did not find evidence for a differential metabolic response to different resuscitation protocols. Lipoprotein concentration appeared inversely correlated to the tolerance of hypoxia, but no biomarkers were identified that were associated with a faster or slower recovery from cardiac arrest. The metabolic profiles could potentially be useful in predicting outcome after hypoxic ischemic insults in newborn pigs and thereby infants and of future guidance for resuscitation strategy.

## Supporting Information

S1 Supplement Correlation matrices at t1 and t3. (PDF)

## Abbreviations

CC: Chest compressions
C:V ratio: Compression to ventilation ratio
CPR: Cardiopulmonary resuscitation
ROSC: Return of spontaneous circulation
TSP: Trimethylsilylpropionate
NMR: Nuclear magnetic resonance spectroscopy
ppm: Parts per million
PCA: Principal component analysis
PLS: Partial least-squares regression
PLS-DA: Partial least-squares regression discriminant analysis
NMC: Number of misclassifications
FC: Fold change
BCAA: Branched-chain amino acids
CPR: Cardiopulmonary resuscitation
