# Supplementary material for: The Role of Plasma and Urine Metabolomics in Identifying New Biomarkers in Severe Newborn Asphyxia: A Study of Asphyxiated Newborn Pigs following Cardiopulmonary Resuscitation
Source: PLoS One. 2016 Aug 16;11(8):e0161123. doi: 10.1371/journal.pone.0161123 (PMC4987056; doi:10.1371/journal.pone.0161123)
Supplement: S1 Supplement — (PDF) [file pone.0161123.s001.pdf]

S1 Supplement: The role of plasma and urine metabolomics in identifying new biomarkers in severe newborn asphyxia

Daniel Sachse, Anne Lee Solevåg, Jens Petter Berg, Britt Nakstad; PLOS ONE

Figure A: Clustered Correlation Map at Time Point t1

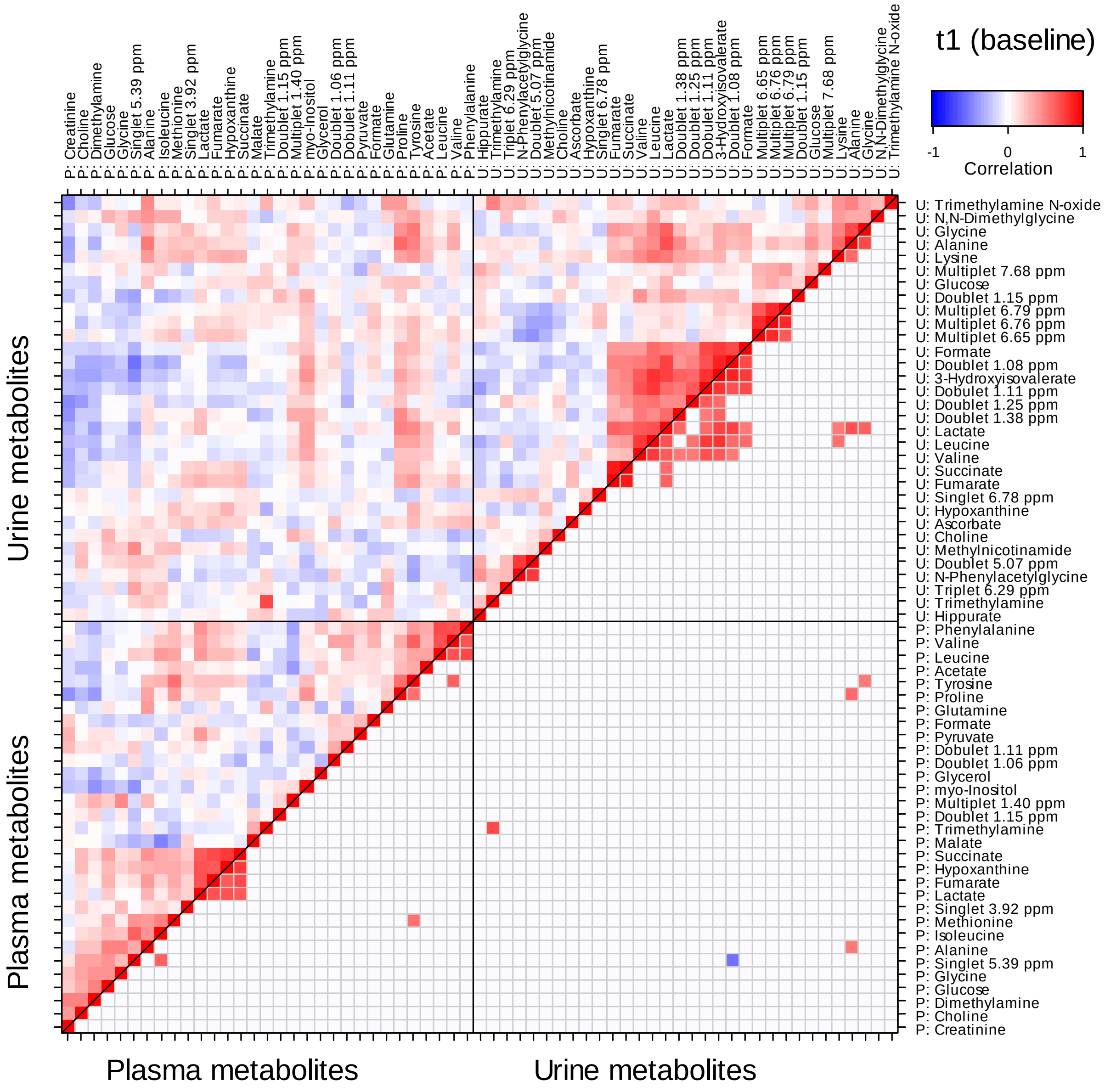

Correlation of plasma and urine metabolites at t1 (under anesthesia and after surgery, immediately before asphyxiation). Positive correlations in red, negative correlations in blue. Few clusters within the two materials and hardly any correlations between materials.

Figure B: Clustered Correlation Map at Time Point t3

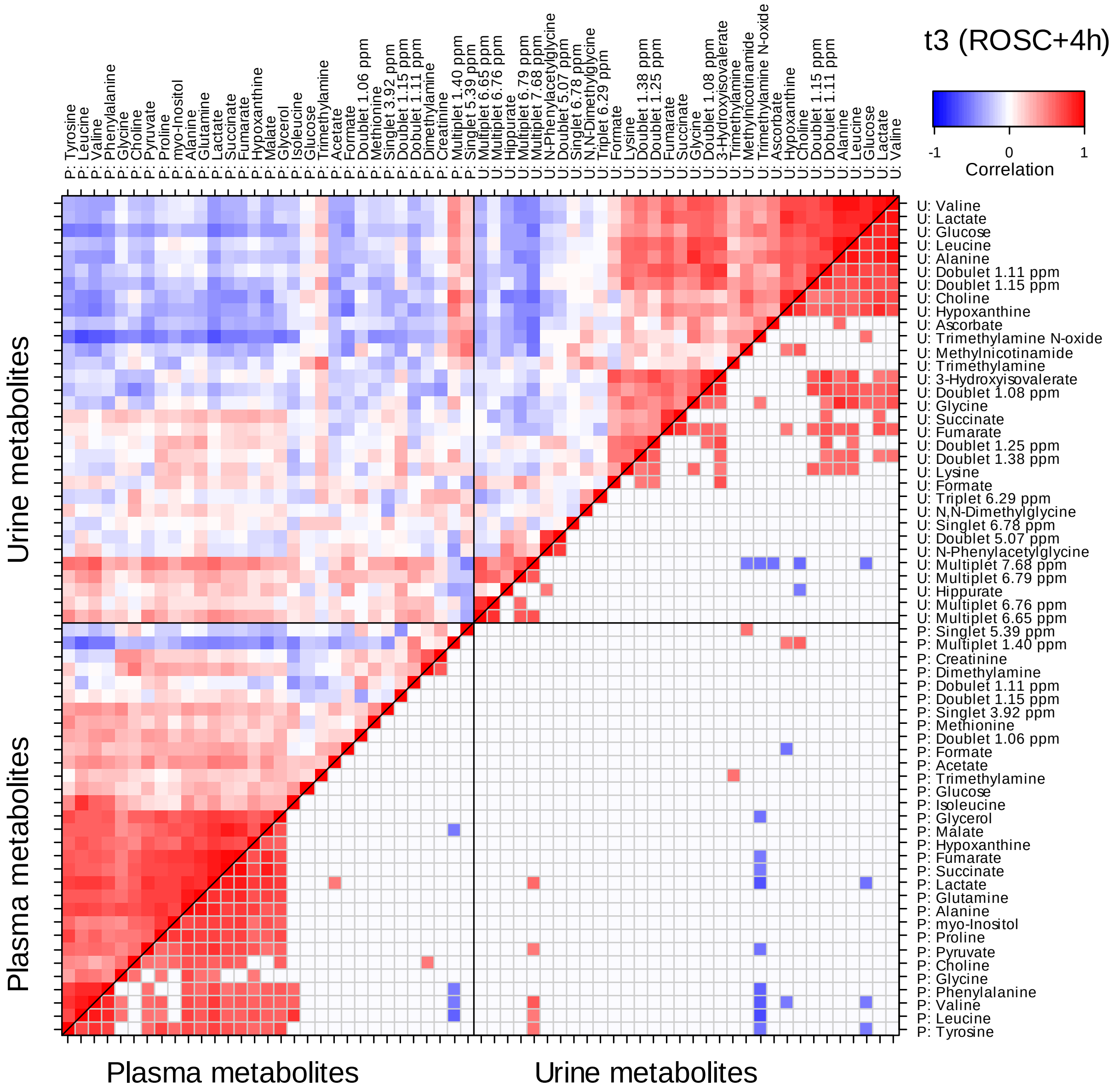

Correlations between plasma and urine metabolites at t3 (4h after return of spontaneous circulation, ROSC). Clusters in both materials are strong. Positive correlations in red, negative correlations in blue. Correlation between the materials is mostly negative, probably due to the excretion/clearance time lag.
